# Glycolate oxidase gene family in *Nicotiana benthamiana*: genome-wide identification and functional analyses in disease resistance

**DOI:** 10.1038/s41598-018-27000-4

**Published:** 2018-06-05

**Authors:** You-Ping Xu, Juan Yang, Xin-Zhong Cai

**Affiliations:** 1 State key laboratory of Rice Biology, Institute of Biotechnology, College of Agriculture and Biotechnology, Zhejiang University, 866 Yu Hang Tang Road, Hangzhou, 310058 China; 2 Centre of Analysis and Measurement, Zhejiang University, 866 Yu Hang Tang Road, Hangzhou, 310058 China

## Abstract

Glycolate oxidase (GOX)-dependent production of H_2_O_2_ in response to pathogens and its function in disease resistance are still poorly understood. In this study, we performed genome-wide identification of *GOX* gene family in *Nicotiana benthamiana* and analyzed their function in various types of disease resistance. Sixteen *GOX* genes were identified in *N*. *benthamiana* genome. They consisted of GOX and HAOX groups. All but two NbGOX proteins contained an alpha_hydroxyacid_oxid_FMN domain with extra 43–52 amino acids compared to that of FMN-dependent alpha-hydroxyacid oxidizing enzymes (NCBI-CDD cd02809). Silencing of three NbGOX family genes *NbHAOX8*, *NbGOX1* and *NbGOX4* differently affected resistance to various pathogens including *Tobacco rattle virus*, *Xanthomonas oryzae* pv. *oryzae* (*Xoo*) and *Sclerotinia sclerotiorum*. Effect of these genes on resistance to *Xoo* is well correlated with that on *Xoo*–responsive H_2_O_2_ accumulation. Additionally, silencing of these genes enhanced PAMP-triggered immunity as shown by increased flg22-elicited H_2_O_2_ accumulation in *NbGOX*-silenced plants. These NbGOX family genes were distinguishable in altering expression of defense genes. Analysis of mutual effect on gene expression indicated that *NbGOX4* might function through repressing *NbHAOX8* and *NbGOX1*. Collectively, our results reveal the important roles and functional complexity of *GOX* genes in disease resistance in *N*. *benthamiana*.

## Introduction

Glycolate oxidase (GOX/GLO) is a crucial enzyme in photorespiration, catalyzing the conversion of glycolate into glyoxylate in peroxisomes with the production of H_2_O_2_^1^. Photorespiration caused by high light intensity, drought and salinity is one of the essential pathways of plant metabolism and resistance to abiotic stress conditions^2^, allowing plant growth in a high oxygen-containing environment^3^. Hydroxy acid oxidase (HAOX) is also located in peroxisomes and has a high similarity to GOX^4^. Therefore, HAOX is recognized as a subgroup of GOX family hereafter.

*GOX* genes have been identified in several plant species including *Arabidopsis thaliana* and rice. *A*. *thaliana* contains five *GOX* family genes, *AtGOX1*-3 and *AtHAOX1*-2^5^. The five members of GOX family possess similar size of amino acid (aa) ranging from 364 to 374 and show high similarity among each other. They all carry an alpha_hydroxyacid_oxid_FMN domain. Rice genome comprises four functional *GOX/GLO* genes and they are differently expressed in rice tissues. Complex interactions exist for GLO isozymes and are coordinately controlled by GLO1 and GLO4^6^. GOX plays roles in a variety of physiological processes. For instance, GOX is required for maize to survive in ambient air^7^. It is involved in drought and salt stress responses^8,9^, and has a strong regulation over photosynthesis, possibly through a feed-back inhibition on Rubisco activase^10^. In addition, a rice GLO appears to affect plant growth^11^.

Moreover, GOX is supposed to play a crucial role in plant disease resistance. The products from a reaction catalyzed by GOX are glyoxylate and H_2_O_2_, both are involved in plant disease resistance. Glyoxylate is an efficient precursor for biosynthesis of oxalate^12^, which is a key molecule in the interactions between plant and pathogens such as *Sclerotinia sclerotiorum* (*Ss*)^13,14^. However, the pathway of oxalate accumulation and regulation seems to be independent of GOX^15^. As an important reactive oxygen species (ROS), H_2_O_2_ functions as an essential signal in the interactions between plant and pathogens^16–19^. Nevertheless, direct evidence supporting a role of *GOX* in plant disease resistance has been mainly reported from the studies in the model plant species *Arabidopsis*, rice and *Nicotiana benthamiana* to date. Arabidopsis mutants of all five *GOX* genes show lowered H_2_O_2_ accumulation, reduced callose deposition, and decreased electrolyte leakage and accordingly lose the nonhost resistance to the bacterial pathogen *Pseudomonas syringae* pv. *tabaci*^5^, demonstrating that all members of the Arabidopsis *GOX* family positively function in this nonhost resistance. However, a study in rice showed the opposite results. Chern *et al*.^11^ reported that knock-down of a rice *GOX* gene *GLO1* using the constitutive and inducible constructs enhanced rice resistance to another bacterial pathogen *Xanthomonas oryzae* pv. *oryzae* (*Xoo*) with increased expression of defense regulator genes *NH1*, *NH3* and *WRKY45*, and thus *GOL1* appeared to play a negative role in this host resistance^11^. In addition, virus induced gene silening (VIGS) analysis of a *GOX* gene in *N*. *benthamiana* indicated its positive role in nonhost resistant to *P*. *syringae* pv. *tomato* and cell death elicited by several *R* genes such as *LOV1*, *RPP8* and *Pto*^5,20^. These results indicate that GOX may play distinct roles in different types of plant disease resistance against various types of pathogens.

In this study, we conducted genome-wide identification of GOX in *N*. *benthamiana* and explored the function of three *NbGOX* genes in various types of resistance. Our results reveal that *GOX* genes play important roles in various types of resistance including PAMP-triggered immunity (PTI), host and nonhost resistance in *N*. *benthamiana* against different pathogens, and members of *NbGOX* gene family employ distinct defense pathways to modulate disease resistance.

## Methods

### Identification of *N*. *benthamiana GOX* family genes

The protein sequences of Arabidopsis GOX family including three AtGOXs and two AtHAOXs were obtained from TAIR and were then used to BLASTp search against *N*. *benthamiana* genome databases in Solanaceae Genomics Network (SGN, http://solgenomics.net/) and University of Sydney (http://sydney.edu.au/science/molecular_bioscience/sites/benthamiana/). All retrieved non-redundant sequences were collected and subjected to domain composition anaysis using Conserved Domain Database (CDD) program (http://www.ncbi.nlm.nih.gov/cdd). Sequences containing a complete alpha_hydroxyacid_oxid_FMN domain were recognized as potential GOX proteins. The *N*. *benthamiana* GOX sequences were aligned with Arabidopsis GOXs using ClustalX 2.01 program^21^ with default settings. The unrooted phylogenetic trees were constructed based on the alignments using MEGA 5.0^22^ with the maximum likelihood (ML) method. The bootstrap analysis was carried out with 1000 replicates. To obtain sequence similarity, GOX family protein sequences in *N*. *benthamiana* and *A*. *thaliana* were compared using MegAlign program of Lasergene software package.

### Virus-induced gene silencing (VIGS) analyses

The coding regions of the *NbGOX* genes are highly conserved. To ensure the specificity of gene silencing, the VIGS target fragment of *NbHAOX8* (Nbv5tr6245008) and *NbGOX1* (NbS00060838g0004.1) were designed to a 203 and 243 bp fragemnt of 3′ UTR region while that of *NbGOX4* (NbS00005125g0015.1) corresponded to a 334 bp fragment of 5′ UTR region. The cloning sites *Bam*HI and *Eco*RI were added to the end of the forward and reverse primers for target fragment amplification, respectively. The PCR product was ligated into pYL156 with *Bam*HI and *Eco*RI, and confirmed by sequencing. The recombinant constructs were transformed into *Agrobacterium tumefaciens* strain GV3101 by electroporating. Agroinfiltration and VIGS analyses were conducted as described^23,24^ except that recombinant pYL156 with insertion of an eGFP fragment instead of an empty pYL156 was used as control to alleviate viral symptom^25^. The first and second true leaves of *N*. *benthamiana* plants at four-leaf stage were infiltrated with the mixture at 1:1 ratio of *Agrobacterium* cell suspensions carrying pTRV1 and recombinant pYL156, respectively, and the agro-inoculated plants were grown in a plant growth chamber at 21 °C with a 16 h/8 h light/dark daily cycle to ensure the silencing efficiency. Three weeks post agro-inoculation, these plants were subjected to phenotype investigation and functional analyses.

### Plant materials for gene exression analyses

For analysis of *NbGOX* gene expression in response to pathogen inoculation, *N*. *benthamiana* were grown in pots in growth chambers at 25 °C with 16 h/8 h light/dark daily cycle. The completely developed leaves of 8-leaf-old plants were inoculated with *Xanthomonas oryzae* pv. *oryzae* (*Xoo*) by infiltrating with bacterial suspensions with an OD_600_ of 0.5. The inoculated leaves were sampled at 0, 1, 6 and 24 h post inoculation (hpi). The harvested samples were immediately frozen in liquid nitrogen and stored at −80 °C until RNA extraction. For analysis of the VIGS efficiency and defense gene expression in VIGS-treated plants, the leaves were collected at 21 d after agro-infiltration.

### Gene expression analysis with quantitative real time PCR

RNA extraction and quantitative real time PCR (qRT-PCR) analyses and consequent statistical data analyses were conducted as described^26^. The primers for amplification of the *NbGOX* genes were designed from the 5′ UTR regions to ensure the gene targeting specificity for single genes. The *N*. *benthamiana* homologs of rice *WRKY45* and *WRKY6*2 which are involved in defense against *Xoo*^27,28^ was identified by BLASTp and phylogenetics analyses. The 18 S rDNA gene was used as a loading check. All primers used in qRT-PCR analyses were listed in Supplementary Table S1.

### Plant disease resistance analysis

Disease resistance was evaluated in the newly, fully expanded leaves of VIGS-treated plants at 21 d after agro-infiltration. Leaf inoculation with *Xoo* was condected as described^29^. *Xoo* was incubated in NA liquid media containing carbenicillin (50 μg/ml). Leaves were inoculated by infiltrating with bacterial suspensions with an OD_600_ of 0.5. The inoculated plants were grown at 25 °C in a growth chamber. Hypersensitive response (HR) was recorded and leaves were classified into three categories according to the percentage of hypersentitive necrosis area over the total inoculated area as described^30^. Bacterial numbers in the inoculated areas were counted as described^29^. Leaf inoculation with *Sclerotinia sclerotiorum* (*Ss*) was performed with mycelial plugs as described^31^, except for that mycelial plugs of 5 mm in diameter were used. Size of disease lesions was measured. For each treatment, at least 6 leaves from 6 silenced plants (one leaf from each plant) were examined. Each experiment was conducted three times independently.

### H_2_O_2_ assays

The H_2_O_2_ in *Xoo*-inoculated leaves was detected *in situ* using diaminobenzidine (DAB) staining as described^19^. The H_2_O_2_ elicited by the PAMP peptide flg22 (400 nM) in leaf discs were quantitatively measured using a Microplate Luminometer (Orion L Microplate Luminometer, Titertek-Berthold, Germany) following previously described protocol^31^. For each treatment, 8 leaves were collected. The experiments were conducted three times independently.

### Statistical data analysis

All experiments were conducted independently three times. Analysis of variance was performed using the SPSS software (Version 19.0, IBM, USA). Significance of the differences between mean values was determined with Student’s *t* test (*p* < 0.05) and Duncan’s multiple range test (DMRT, *p* < 0.05).

## Results

### Identification of *GOX* family genes in *N*. *benthamiana* genome

To identify *GOX* family genes in *N*. *benthamiana* genome, BLASTp search was performed for five Arabidopsis GOX family protein sequences including three AtGOXs and two AtHAOXs against the *N*. *benthamiana* genome databases in SGN (http://solgenomics.net/) and University of Sydney (http://sydney.edu.au/science/molecular_bioscience/sites/benthamiana/), respectively. Consequently, 10 and 22 candidate sequences were retrieved from the SGN database and University of Sydeny database, respectively. Further domain composition analysis using CDD program (http://www.ncbi.nlm.nih.gov/cdd) demonstrated that 9 and 7 candidate genes from the SGN database and Sydeny database, respectively, contained an alpha_hydroxyacid_oxid_FMN domain, a characteristic domain for all Arabidopsis GOX proteins. These 16 sequences were recognized as GOX family genes. Sequence alignment analysis indicated that 6 out of 7 sequences from University of Sydney database showed high similarity to *AtHAOXs*, while 9 sequences from SGN database displayed similarity to both *AtGOXs* and *AtHAOXs*. It’s notable that one sequence from SGN (NbS00018477g0026.1) comprised of 836 aa with two alpha_hydroxyacid_oxid_FMN domains. Further protein sequence alignment of this candidate with AtGOXs suggested that it was a fusion of two *NbHAOXs* with 337 aa and 499 aa, respectively (Table 1, Supplementary Fig. S1).

**Table 1:** The *GOX* gene family in *Nicotiana benthamiana*.

Gene	Locus number^*^	Protein size (aa)	Domain
NbGOX1	NbS00060838g0004.1	368	alpha_hydroxyacid_oxid_FMN
NbGOX2	Nbv5tr6239807	368	alpha_hydroxyacid_oxid_FMN
NbGOX3	NbS00022202g007.1	333	alpha_hydroxyacid_oxid_FMN
NbGOX4	NbS00005125g0015.1	356	alpha_hydroxyacid_oxid_FMN
NbGOX5	NbS00025736g0004.1	356	alpha_hydroxyacid_oxid_FMN
NbGOX6	NbS00024535g0016.1	349	alpha_hydroxyacid_oxid_FMN
NbGOX7	NbS00043092g0003.1	384	alpha_hydroxyacid_oxid_FMN
NbHAOX1	NbS00018477g0026.1-(338-836)	499	alpha_hydroxyacid_oxid_FMN
NbHAOX2	Nbv5tr6233382	363	alpha_hydroxyacid_oxid_FMN
NbHAOX3	NbS00001920g0010.1	294	alpha_hydroxyacid_oxid_FMN
NbHAOX4	Nbv5tr6245007	363	alpha_hydroxyacid_oxid_FMN
NbHAOX5	Nbv5tr6211426	302	alpha_hydroxyacid_oxid_FMN
NbHAOX6	NbS00018477g0026.1-(1-337)	337	alpha_hydroxyacid_oxid_FMN
NbHAOX7	Nbv5tr6200247	364	alpha_hydroxyacid_oxid_FMN
NbHAOX8	Nbv5tr6245008	363	alpha_hydroxyacid_oxid_FMN
NbHAOX9	NbS00010324g0011.1	363	alpha_hydroxyacid_oxid_FMN
NbHAOX10	Nbv5tr6245006	364	alpha_hydroxyacid_oxid_FMN

*Genes with a number starting with NbS are from the database of Solanaceae Genomics Network while those starting with Nbv5 are from the database of University of Sydney.

**Figure 1:**
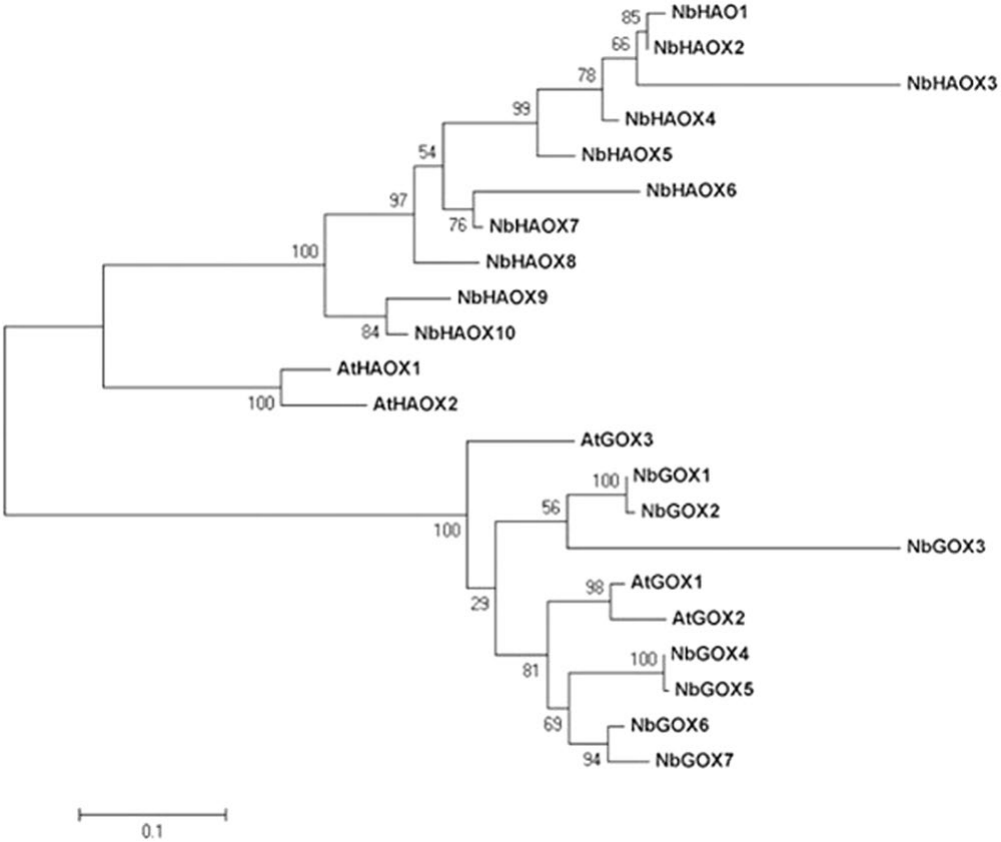
Phylogenetic tree of GOX family proteins in *Nicotiana benthamiana* and *Arabidopsis thaliana*. The tree was created using Clustalx program by maximum liklihood method with bootstrap of 1000 in MEGA5. The protein sequences are listed in Table 1 and Supplementary Fig. S1.

Bioinformatics analyses predicted that NbGOXs were basic proteins with a size of 294 aa (*NbHAOX3)* ~480 aa (*NbHAOX1*) that was dominated by about 360 aa (Table 1). It is noteworthy that domain prediction analysis using NCBI-CDD indicated that the alpha_hydroxyacid_oxid_FMN domain of all the identified *N*. *benthamiana* and Arabidopsis GOX sequences except NbHAOX3 and NbHAOX6 contained an extra fragment in the middle compared with that of FMN-dependent alpha-hydroxyacid oxidizing enzymes deposited in NCBI-CDD (cd02809). The size of this extra fragment was 43 aa for HAOXs while 46 aa for GOXs, expect AtGOX2 (AT3G14415.2) whose was 52 aa (Supplementary Fig. S1). Function of this extra fragment was unclear. Sequences of the NbGOX family showed a high similarity within groups but displayed much lower similarity between groups. The protein sequence similarity was 73.0–99.7% within the NbGOX group while 74.1–98.9% within NbHAOX group, whereas only 45.4–60.9% between the two groups (Supplementary Fig. S2). CDD analyses demonstrated that all Arabidopsis and *N*. *benthamiana* GOX family proteins carried 3 putative catalytic residues, and most of them harbored 9 active sites, 5 subtrate binding sites and 7 FMN binding sites. The extra fragment appeared after the second putative catalytic residues (Supplementary Fig. S1). To better reflect the orthologous relationship between the *N*. *benthamiana* and Arabidopsis *GOX* genes, we named the *N*. *benthamiana* members in accordance with the phylogeny and sequence similarity to individual Arabidopsis *GOXs* (Table 1, Fig. 1).

### Phylogenetic relationship between *N*. *benthamiana* and Arabidopsis *GOXs*

The full-length amino acid sequences of *N*. *benthamiana* and Arabidopsis GOXs were subjected to phylogenetic analysis. The ML phylogenetic tree showed that alpha_hydroxyacid_oxid_FMN domain-containing proteins were divided into two major groups, group GOX and group HAOX (Fig. 1). All NbGOXs were from SGN database except NbGOX2, while NbHAOX sequences were from both SGN and University of Sydney databases. HAOX sequences from *N*. *benthamiana* and Arabidopsis were separated into two clades, while GOX sequences from the two species not, suggesting that HAOXs from the two species are more distant evolutionarily compared with GOXs.

### *NbGOX* genes are differentially expressed in response to the nonhost pathogen *Xoo* in *N*. *benthamiana*

**Figure 2:**
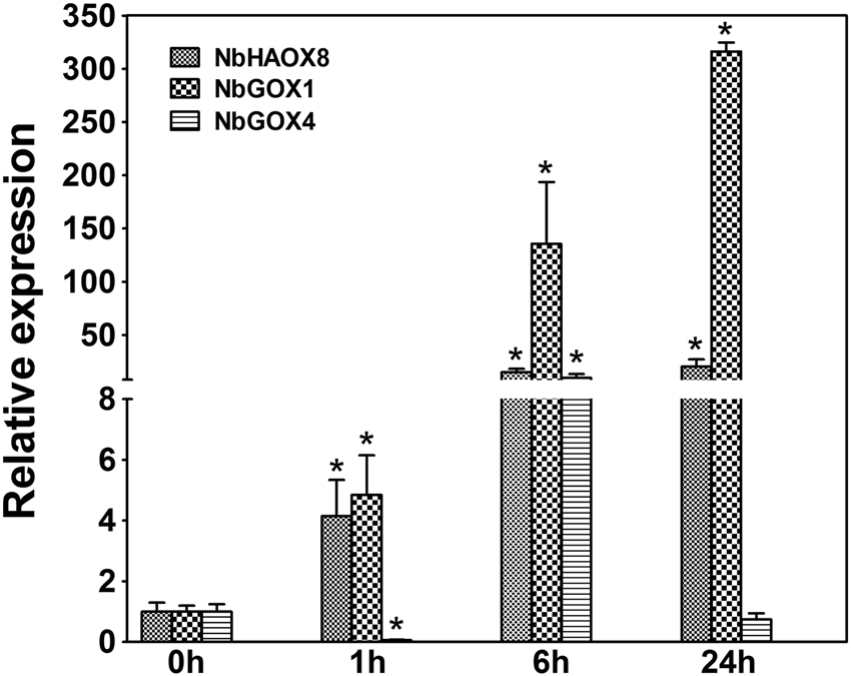
Expression of three NbGOX family genes *NbHAOX8*, *NbGOX1* and *NbGOX4* in *N*. *benthamiana* in response to *Xoo* inoculation. Gene expression was analyzed by qRT-PCR with gene-specific primers listed in Supplementary Table S1. Significant difference between expression values at various timepoints and that at 0 hpi is indicated as a “*”(*p* < 0.05, Student’s *t* test). Data represent the mean ± SE of three independent experiments.

Three *GOX* family genes, *NbHAOX8*, *NbGOX1* and *NbGOX4*, each representing a *N*. *benthamiana* homolog of three clades of AtGOX family (AtHAOX1/AtHAOX2, AtGOX1/AtGOX2, and AtGOX3) in the ML phylogenetic treee, were selected for the analysis of their expression in response to inoculation with the nonhost pathogen *Xoo*. qRT-PCR analysis using gene specific primers (Supplementary Table S1) demonstrated that the expression patterns were distinct between the three selected *GOX* family genes in response to *Xoo*. Expression of *NbHAOX8* and *NbGOX1* was induced as early as 1 h post inoculation (hpi) and consistently increased henceforth but with different magnitude, by 20.2- and 316.2-fold, respectively, at 24 hpi compared with that at 0 hpi, while expression of *NbGOX4* was strongly suppressed at 1 hpi, significantly induced by 9.7-fold at 6 hpi, and then dropped to basal level at 24 hpi compared with that at 0 hpi (Fig. 2). This result indicates that these *GOX* family genes may play different roles in nonhost defense responses to *Xoo* in *N*. *benthamiana*.

### Silencing of *N*. *benthamiana GOX* genes significantly reduces resistance to viral pathogen TRV

**Figure 3:**
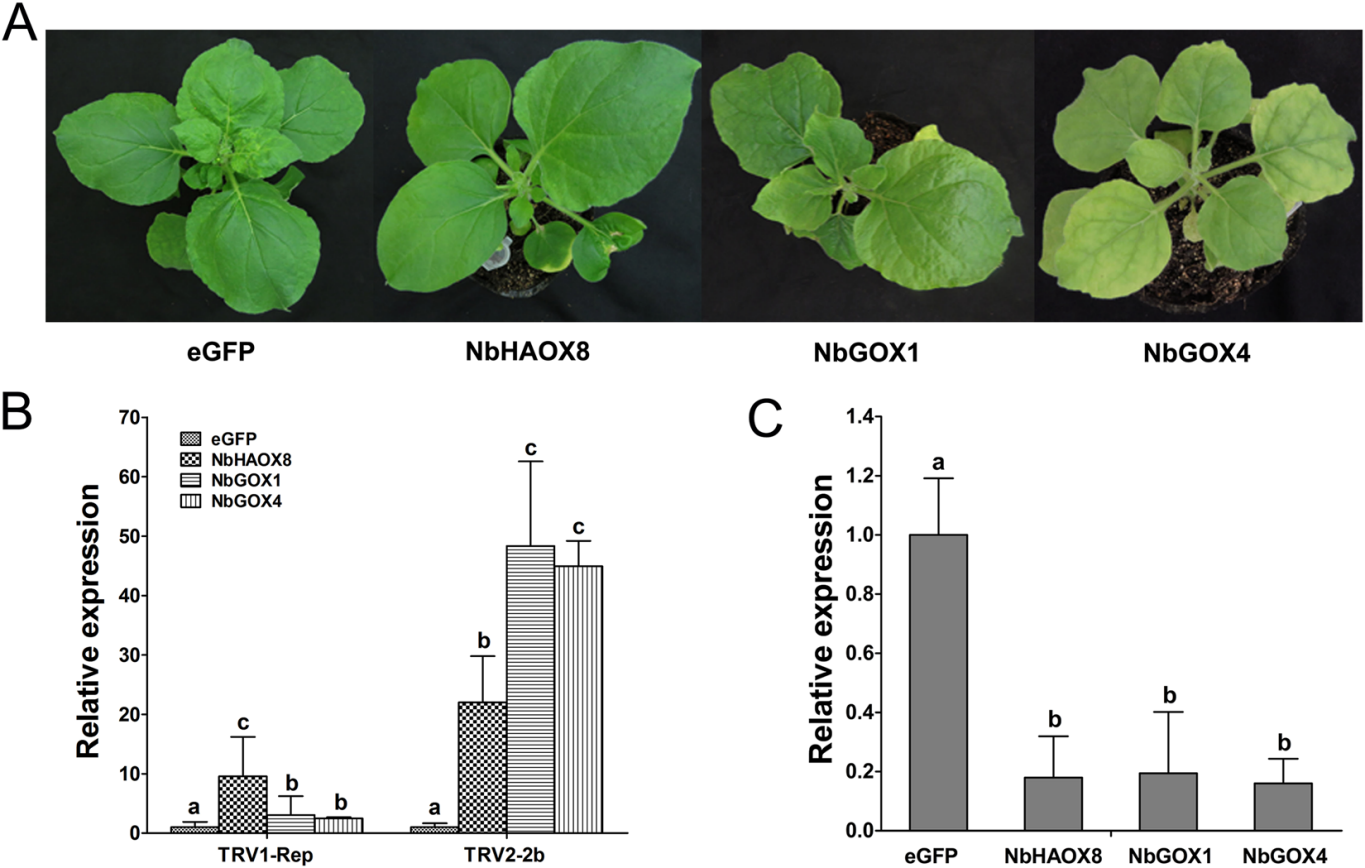
Silencing of *NbHAOX8*, *NbGOX1* and *NbGOX4* reduced resistance to TRV in *N*. *benthamiana*. (**A**) Phenotypes of the gene-silenced plants. Gene silencing analyses were performed using TRV-based vectors. Photographs were taken at 3 weeks post agro-infiltration. (**B**) Detection of transcripts of the TRV1 replicase and TRV2 2b genes in gene-silenced plants by qRT-PCR. (**C**) Evaluation of gene silencing efficiency. Gene expression was examined by qRT-PCR. Significant difference between expression values in gene-silenced plants and eGFP control plants is indicated as different lowercase letters (*P* < 0.05, DMRT).

To probe the function of *NbGOX* family genes, VIGS analyses were performed individually for *NbHAOX8*, *NbGOX4* and *NbGOX1*. To ensure the specificity of gene silencing, the primers used for making VIGS construct were designed at the gene specific UTR sequences of these single genes (Supplement Table 1). The VIGS fragments were ligated into the TRV silencing vector pYL156 for silencing analyses, and non-silenced eGFP fragment-inserted pYL156 vector was used as a negative control^25^. Three weeks after silencing treatment, plants of eGFP control showed no or only very mild mosaic symptoms in some leaves, while those VIGS-treated for *NbGOX4*, *NbHAOX8* and *NbGOX1* showed different degree of yellowing symptoms and/or retarded growth. *NbHAOX8*-silenced plants exhibited no obvious yellowing symptoms but showed retarded growth with less leaves; *NbGOX1*-silenced plants displayed mild yellowing symptoms and retarded growth with less leaves, while *NbGOX4*-silenced plants exhibited strong yellowing symptoms but showed no retarded growth (Fig. 3A). This result suggests that these *NbGOX* family genes may slightly affect plant growth.

To examine effect of VIGS treatment on resistance to the virus TRV, accumulation of viral component gene transcripts in VIGS treated plants and eGFP control plants was comparatively analyzed by qRT-PCR. Result showed that transcripts of TRV1 replicase and TRV2 2b genes accumulated 9- and 22-fold higher in *NbHAOX8*-silenced plants while over 3- and 44-fold in *NbGOX1*- and *NbGOX4*-silenced plants, respectively, compared with eGFP-control plants (Fig. 3B). This result indicates that these *GOX* family genes may positively affect plant resistance to TRV.

To clarity whether the phenotypes were attributed to silencing of these *NbGOX* family genes, silencing efficiency of the VIGS treatments were monitored by detecting the transcripts of these genes in the agro-infiltrated plants with qRT-PCR. qRT-PCR results showed that the transcripts of all three *NbGOX* family genes in the agro-infiltrated plants dropped to lower than 20% of that in eGFP-control plants (Fig. 3C). These results demonstrate that all three genes in the VIGS treated plants had been efficently silenced. Collectively, *N*. *benthamiana GOX* family genes *NbHAOX8*, *NbGOX1* and *NbGOX4* play a positive role in resistance to TRV.

### Silencing of *N*. *benthamiana GOX* genes alters nonhost resistance to *Xoo*

**Figure 4:**
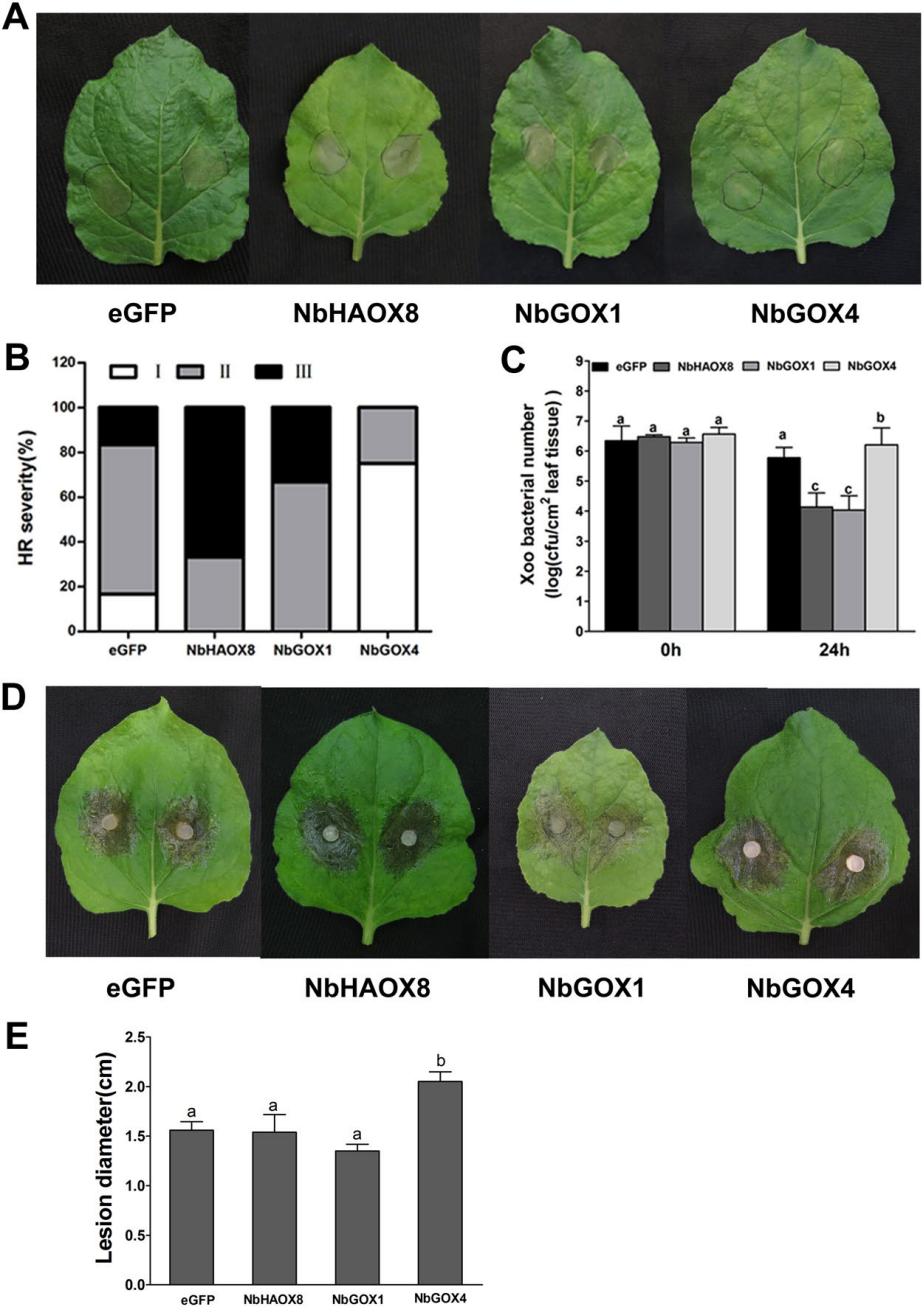
Silencing of *NbHAOX8*, *NbGOX1* and *NbGOX4* differently affected resistance to *Xoo* and *Sclerotinia sclerotiorum* in *N*. *benthamiana*. Disease resistance was evaluated in the newly, fully expanded leaves of VIGS-treated plants at 21 d after agro-inoculation. For each treatment, at least 6 leaves from 6 silenced plants (one leaf from each plant) were examined. Each experiment was conducted three times independently. (**A**) Symptoms of *Xoo*-induced HR in the gene-silenced plants. Photographs were taken at 18 hpi. (**B**) Severity of *Xoo*-induced HR in the gene-silenced plants at 18 hpi. HR is classified based on the intensity of cell death in inoculated areas: I, HR area smaller than 30% of infiltrated leaf area; II, HR area larger than 30% but smaller than 90% of infiltrated leaf area; III, HR area larger than 90% of the infiltrated leaf area. HR severity is indicated as the percentage of leaves exhibiting HR over total infiltrated leaves. (**C**) Statistical analysis of *Xoo* bacterial number in the infiltrated areas at 0 and 24 hpi. Data were analyzed using SPSS. Significant difference is indicated as different lowercase letters (*P* < 0.05, DMRT). (**D**) Disease symptoms of the gene-silenced plants after inoculation with *Ss*. Photographs were taken at 26 hpi. (**E**) Statistical analysis of lesion diameter. Significant difference of lesion diameter is indicated as lowercase letters (*P* < 0.05, DMRT).

To understand the role of *NbGOX* family genes in nonhost resistance, the silenced *N*. *benthamiana* plants were inoculated with the nonhost pathogen *Xoo*. The HR necrosis of leaves of *NbHAOX8-* and *NbGOX1-*silenced plants was obviously more severe while that of the *NbGOX4*-silenced plants was weaker than that of the eGFP-control plants (Fig. 4A). At 18 hpi, 67% and 33% leaves of *NbHAOX8-* and *NbGOX1-*silenced plants, respectively, exhibited complete or nearly complete HR necrosis (type III), this percentage for the eGFP-control plants was 17%, while no leaf of *NbGOX4*-silenced plants displayed this type of HR (Fig. 4B). Further, bacterial number counting assay demondtrated that *Xoo* bacterial number in inoculated areas of *NbHAOX8-* and *NbGOX1-*silenced plants was reduced by near 2 orders of magnitude while that of *NbGOX4*-silenced plants increased by 0.4 orders of magnitude compared with that of the eGFP-control plants at 24 hpi (Fig. 4C). Together, these results indicate that *NbHAOX8* and *NbGOX1* play a negative role while *NbGOX4* plays a positive role in nonhost resistant to *Xoo*.

### Silencing of *N*. *benthamiana GOX* genes changes host resistance to *Sclerotinia sclerotiorum*

To explore the role of *NbGOX* genes in resistance to fungal pathogen, the silenced *N*. *benthamiana* plants were inoculated with the necrotrophic fungal pathogen *Ss*. *NbHAOX8-*silenced plants displayed similar size of lesions to the eGFP-control plants, *NbGOX1-*silenced plants exhibited smaller lesions, while *NbGOX4*-silenced plants showed larger lesions than the eGFP-control plants (Fig. 4D). The average lesion size of *NbHAOX8*-, *NbGOX1*- and *NbGOX4*-silenced plants was 1.54, 1.35 and 2.05 cm in diameter, respectively, at 24 hpi (Fig. 4E), which was in turn statistically similar to, smaller and larger than the lesion size of the eGFP-control plants (1.56 cm on average) (Fig. 4E). This suggests that *NbGOX1* may play a negative role while *NbGOX4* plays a positive role in basal resistance to the necrotrophic pathogen *Ss*. However, *NbHAOX8* might not contribute to this basal resistance.

### Silencing of *N*. *benthamiana GOX* genes modulates pathogen- and PAMP-elicited reactive oxygen species

**Figure 5:**
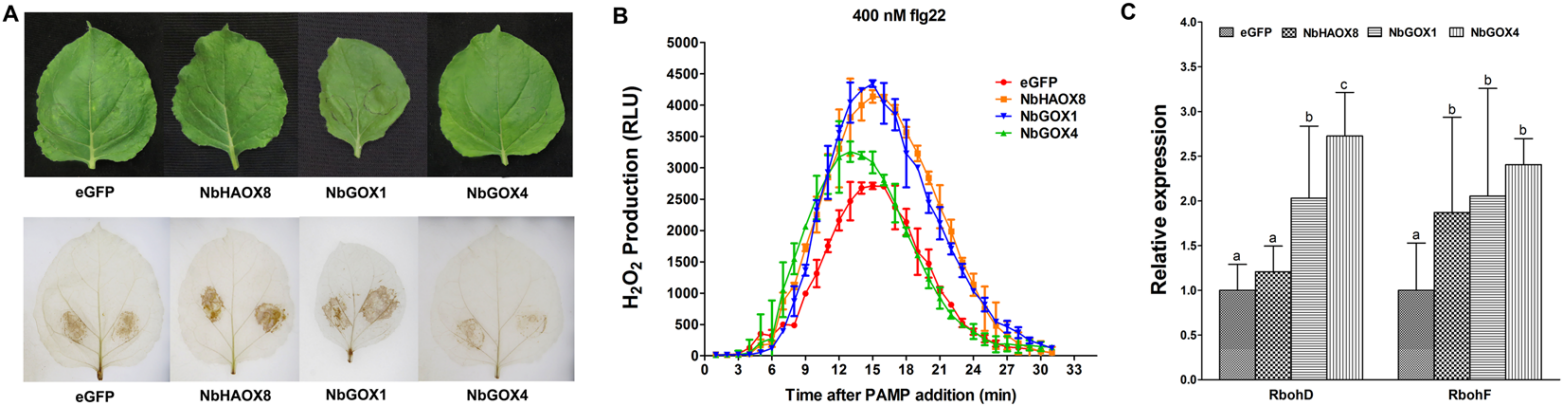
Silencing of *NbHAOX8*, *NbGOX1* and *NbGOX4* differently modulated H_2_O_2_ in *N*. *benthamiana*. (**A**) Detection of *Xoo*-elicited H_2_O_2_ in gene-silenced plants at 12 hpi by DAB staining. No HR occurred in these plants (upper panel). (**B**) Dynamics of H_2_O_2_ accumulation in response to flg22 (400 nM) elicitation in leaves of gene-silenced plants. Flg22-triggered H_2_O_2_ bursts were measured using luminol-based assay in leaf discs. Data are shown as relative luminal units (RLU) and represent the mean ± SE of three independent experiments. (**C**) Effect of silencing of *NbHAOX* and *NbGOX* genes on expression of *RbohD* and *RbohF* genes. Gene expression was analyzed by qRT-PCR. Data represent the mean ± SE of three independent experiments. Significant difference between expression values is indicated as lowercase letters (*P* < 0.05, DMRT).

ROS is essential in resistance to *Xoo* and *Ss* in *N*. *bemthaniana*^13,19^. To probe the contribution of ROS to the *NbGOX*-mediated resistance, H_2_O_2_ accumulation in silenced and control plants was compared. DAB staining assay showed that H_2_O_2_ accumulated more highly in *Xoo*-inoculated areas of *NbHAOX8-* and *NbGOX1-*silenced plants while more weakly in those of *NbGOX4*-silenced plants than in those of the eGFP-control plants at 12 hpi, a timepoint when HR had not yet occurred (Fig. 5A). This result indicates that *NbGOX* family genes might modulate ROS accumulation in resposne to pathogen thereby alter resistance.

The ROS accumulation is also a hallmark of pathogen-associated molecular pattern (PAMP)-triggeted immunity (PTI). To examine the role of *NbGOX* genes in PTI, effect of silencing of *NbGOX* genes on PAMP-elicited ROS accumulation was monitored. Flg 22 (400 nM) were applied to leaf discs (4 mm in diameter) and the dynamics of H_2_O_2_ accumulation was measured. Quantitative detection by luminol-based approach demonstrated that, for the eGFP-control plants, H_2_O_2_ increased rapidly and peaked at 15 min in response to flg22 and then decreased quickly to reach the basal level within about 30 min. However, *NbGOX*-silenced plants showed different flg22-elicited H_2_O_2_ accumulation compared with the eGFP-control plants. Upon flg22 supply, luminol-based signal repersenting H_2_O_2_ accumulation in *NbHAOX8-*, *NbGOX1-* and *NbGOX4-*silenced plants culminated at 4139, 4342 and 3257 RLU, respectively, which was stronger than that in the eGFP-control plants (2710 RLU) (Fig. 5B). This result implies that *NbHAOX8* and *NbGOX1* might play a negative role in flg22-triggered PTI and *NbGOX4* might function similarly but to a less level.

### Silencing of *N*. *benthamiana GOX* genes does not significantly change expression of NADPH oxidases genes

Plant *Rboh* gene family encodes subunits of NADPH oxidase. This family, especially RbohD and RbohF, is an important source to generate ROS during the interactions between plant and pathogens^16,32^. To clarify whether *NbGOX* genes modulate ROS accumulation via *Rboh* genes, effect of *NbGOX* gene silencing on the expression of *RbohD* and *RbohF* genes was examined. qRT-PCR analysis showed that the expression of *RbohD* and *RbohF* genes in the three *NbGOX*-silenced plants was higher to different extent than that in the eGFP control plants (Fig. 5C). In *NbHAOX8*-silenced plants, expression of *RbohD* and *RbohF* genes was 1.2-fold and 1.8-fold, respectively, as high as that in control plants. In *NbGOX1*-silenced plants, expression of both *Rboh* genes was about 2-fold as high as that in control plants; while in *NbGOX4*-silenced plants, expression of *RbohD* and *RbohF* genes was 2.7-fold and 2.4-fold, respectively, as high as that in control plants (Fig. 5C). Influence of change of the *Rboh* gene expression at these levels (less than 3-fold) on ROS generation and accumulation awaits further elucidation.

### Silencing of *N*. *benthamiana GOX* genes in plants strongly and differently alters expression of defense-related genes

**Figure 6:**
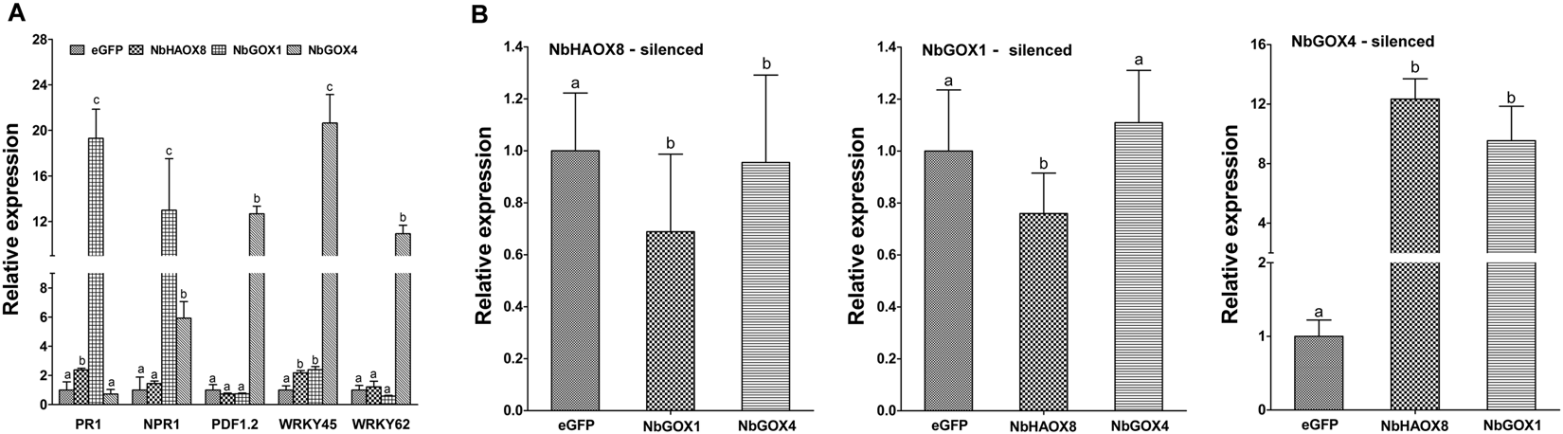
Silencing of *NbHAOX8*, *NbGOX1* and *NbGOX4* differently altered defense related genes and other member genes of *NbGOX* family in *N*. *benthamiana*. (**A**) Expression profiles of *PR1*, *NPR1*, *PDF1*.2, *WRKY45* and *WRKY62* genes in gene-silenced plants. (**B**) Mutual gene expression modulation of *NbHAOX8*, *NbGOX1* and *NbGOX4*. Expression of the other two genes in single GOX gene-silenced plants was examined by qRT-PCR. Data represent the mean ± SE of three independent experiments. Significant difference between expression values is indicated as different lowercase letters (*P* < 0.05, DMRT).

To further study the molecular mechanisms underlying *NbHAOX8*, *NbGOX1* and *NbGOX4*-mediated plant resistance, we monitored the expression of genes involved in defense against pathogens including *Xoo*, such as *PR1*, *NPR1*, *WRKY45* and *WRKY6*2^27,28^, in gene-silenced plants. The qRT-PCR analysis showed that expression of all the five genes was not obviously changed in *NbHAOX8*-silenced plants compared to that in the eGFP control plants. However, expression of these genes was significantly altered in the other two *NbGOX*-silenced plants. Compared with the eGFP control plants, expression of *PR1*, *NPR1*, and *WRKY45* increased by 19-, 13- and 2.4-fold, respectively, whereas expression of *PDF1*.2 and *WRKY6*2 decreased by 26% and 42% in *NbGOX1*-silenced plants. In *NbGOX4*-silenced plants, expression of *NPR1*, *PDF1*.2, *WRKY45* and *WRKY62* was enhanced by 6-, 12-, 20- and 10-fold, respectively, while expression of *PR1* was reduced by 38% (Fig. 6A). These demonstrate that silencing of *NbGOX1* strongly enhances expression of *PR1* and *NPR1* while obviously reduces that of *WRKY62*; silencing of *NbGOX4* strongly increases expression of *NPR1*, *PDF1*.*2*, *WRKY45* and *WRKY62* while decreases that of *PR1*, whereas silencing of *NbHAOX8* does not obviously alter expression of all five checked genes. This indicates that *NbGOX1*-dependent resistance may involve salicylic acid (SA) and *WRKY62*-mediated pathways; *NbGOX4*-dependent resistance may relate to jasmonic acid (JA), *WRKY45*- and *WRKY62*-mediated pathways, while *NbHAOX8*-dependent resistance may be linked to pathways unrelated with these checked genes.

### *NbGOX4* might function through suppressing *NbHAOX8* and *NbGOX1*

*NbHAOX8*, *NbGOX1* and *NbGOX4* are all involved in ROS and resistance, which prompted us to examine the mutual influence of these genes. We analyzed the effect of silencing of one gene on expression of the remaining two genes. Result showed that silencing of *NbHAOX8* or *NbGOX1* individually did not strikingly alter expression of the other two genes, while silencing of *NbGOX4* strongly enhanced expression of *NbHAOX8* and *NbGOX1* genes by approximately 12- and 9-fold, respectively (Fig. 6B). This result implies that *NbGOX4* might function through suppressing *NbHAOX8* and *NbGOX1*.

## Discussion

### The alpha_hydroxyacid_oxid_FMN domain of GOX family in plants

In this study, we identified 16 GOX family genes including 7 GOX and 9 HAOX genes in *N*. *benthamiana* genome (Table 1). Both GOX and HAOX proteins possess an alpha_hydroxyacid_oxid_FMN domain and have a similar size (Table 1, Supplementary Fig. S1), yet, they clustered in distinct groups in the ML phylogenetic tree (Fig. 1). Interestingly, except NbHAOX3 and NbHAOX6, the alpha_hydroxyacid_oxid_FMN domain of all *N*. *benthamiana* GOX family sequences identified in this study and Arabidopsis GOX family sequences identified previously^5^ contained an extra fragment after the second putative catalytic residues compared with that of FMN-dependent alpha-hydroxyacid oxidizing enzymes deposited in NCBI-CDD (cd02809). It is noteworthy that the size of this extra fragment was conserved within HAOX and GOX subfamilies (Supplementary Fig. S1). According to the annotation of cd02809, family of FMN-dependent alpha-hydroxyacid oxidizing enzymes occurs in both prokaryotes and eukaryotes. Members of this family include flavocytochrome b2 (FCB2), glycolate oxidase (GOX), lactate monooxygenase (LMO), mandelate dehydrogenase (MDH), and long chain hydroxyacid oxidase (LCHAO). Containing an extra fragment in the middle of the alpha_hydroxyacid_oxid_FMN domain may be a characteristic feature of plant GOX group of the whole family of FMN-dependent alpha-hydroxyacid oxidizing enzymes. Function of this extra fragment in GOX enzymes remains to be explored.

### Function of GOX in disease resistance in *N*. *benthamiana*

Role of *GOX* in plant disease resistance is seldom studied to date. Attention on this area is not attracted untill recently Rojas *et al*.^5^ found that an *NbGOX* gene played a positive role in nonhost resistant to *P*. *syringae* pv. *tomato* when they conducted VIGS screening for resistance regulatory genes in *N*. *benthamiana*^5^. They further revealed that all five members of the Arabidopsis *GOX* family positively function in nonhost resistance to *P*. *syringae* pv. *tabaci*. Meanwhile, Chern *et al*.^11^ silenced a rice *GOX* gene *GLO1* in rice by using the constitutive and inducible construct, and found that *GLO1* appeared to play a negative role in rice resistance to *Xoo*^11^. In addition, Gilbert and Wolpert^20^ reported a *GOX* gene in *N*. *benthamiana* played a positive role in cell death triggered by *R* genes such as *LOV1*, *RPP8* and *Pto*^20^. In the present study, we analysed function of three representative GOX family genes *NbHAOX8*, *NbGOX1* and *NbGOX4* in *N*. *benthamiana* through VIGS. We found that all three genes play a positive role in resistance to TRV (Fig. 3), but they differently contribute to nonhost resistant to *Xoo* and basal resistant to the necrotrophic pathogen *Ss* (Fig. 4). Furthermore, we for the first time examined the role of GOX in PTI through detecting the flg22-elicited ROS accumulation, and found that *NbHAOX8* and *NbGOX1* might play a stronger negative role while *NbGOX4* play a weaker negative role in flg22-triggered immunity (Fig. 5B). Our results clearly show that unlike Arabidopsis GOXs, which although have divergent functions in the photorespiratory pathway^33^, all function similarly and additively in nonhost resistance^5^, members of GOX family in *N*. *benthamiana* play distinct roles in resistance to the same pathogens and in immunity triggered by the same elicitors. Moreover, one GOX family gene in *N*. *benthamiana* might play distinct roles in different types of resistance (host basal resistance, nonhost resistance and PTI) against different types of pathogens. Collectivelly, these reveal that GOX family genes are involved in various types of disease resistance and this function of GOX family genes may differ up to plant species, gene members, types of resistance and pathogens/elicitors.

### Functional mechanisms of GOX in disease resistance in *N*. *benthamiana*

It is conceivable that the most direct mechanism underlying GOX-mediated resistance should be modulating H_2_O_2_ during the interactions between plant and pathogen. Our DAB staining results clearly showed that silencing of *NbHAOX8* and *NbGOX1* increased while silencing of *NbGOX4* reduced *Xoo*-elicited H_2_O_2_ accumulation, demonstrating that *NbHAOX8* and *NbGOX1* play a negative role while *NbGOX4* plays a positive role in this H_2_O_2_ accumulation (Fig. 5A). Effect of these genes on *Xoo*–responsive H_2_O_2_ accumulation was well correlated with that on resistance to *Xoo* (Fig. 4). Considering that H_2_O_2_ is indisapensible for the nonhost resistance to *Xoo* in *N*. *benthamiana*^19^, this result implies that these GOX family genes alter resistance via modulating H_2_O_2_ accumulation. Moreover, examination of the role of GOX in PAMP elicited ROS demonstrated that *NbHAOX8* and *NbGOX1* and probably also *NbGOX4* might play a negative role in flg22-elicited ROS (Fig. 5B). Our results reveal that GOX family regulates H_2_O_2_ accumulation not only during nonhost resistance nut also during PTI. In this context, it is interesting that rice GLO1 was found to interact with a glutaredoxin protein^34^. How NbGOX family proteins coordinate with other components to regulate cellular redox awaits further dissection.

A well-known mechanism to generate ROS during the interactions between plant and pathogen is from NADPH oxidase encoded by *Rboh* genes in plants, especially *RbohD* and *RbohF*^16,18,32,35^. It was reported that, In Arabidopsis, GOX and Rboh function independently in regulating ROS during nonhost resistance^5^. Whether this is also the case for GOX in *N*. *benthamiana* deserves to be elucidated. We monited the expression of *RbohD* and *RbohF* in GOX-silenced plants, and found that silencing of three *NbGOX* family genes increased expression of the two *Rboh* genes to 1.2~2.7-fold as high as that in control plants (Fig. 5C). It is unclear whether change of the *Rboh* gene expression at this level (less than 3-fold) significantly influences ROS generation and accumulation. Therefore, it remains to be clarified whether *NbGOX* genes modulate ROS accumulation through altering *RbohD* and *RbohF* gene expression.

In addition to differently modulate ROS accumulation, the three *NbGOX* family genes *NbHAOX8*, *NbGOX1* and *NbGOX4* promote different defense signaling pathways. Our qRT-PCR results showed that silencing of *NbGOX1* strongly increased the expression of *PR1* and *NPR1*, silencing of *NbGOX4* significantly promoted the expression of *NPR1*, *PDF1*.*2*, *WRKY45* and *WRKY62*, while silencing of *NbHAOX8* did not obviously alter expression of all 5 checked genes (Fig. 6A). *PR1* and *PDF1*.*2* are well known marker genes of SA and JA defense signaling pathways, while *WRKY45* and *WRKY62* are important defense regulatory genes involved in resistance to a variety of pathogens including *Xoo*^27,28,36–39^. Our results indicate that *NbGOX1* negatively regulate SA pathway, *NbGOX4* negatively modulate JA and *WRKY45*- and *WRKY62*-mediated pathways; while *NbHAOX8* might involve pathways unrelated with these checked genes. These differences may partially explain why these *NbGOX* family genes differently modulate the disease resistance. Additionally, our result is different from what has been reported for Arabidopsis HAOX2, which activates the SA pathway and is mediated by PAD4, NPR1, and PR-1, based on the comparative expression of these genes in Col-0 and *haox2* mutant after inoculation with the nonhost pathogen *P*. *syringae* pv. *tabaci*^5^. This might relect the difference between constitutive and pathogen responsive expression of GOX family genes. Alternatively, *GOX* family genes in Arabidopsis and *N*. *benthamiana* might have distinct mechanisms to regulate plant defense against pathogens.

Finally, our results on analysis of the mutual effect of the three *NbGOX* family genes on gene expression showed that silencing of *NbHAOX8* or *NbGOX1* individually did not obviously alter expression of the other two genes, while silencing of *NbGOX4* strongly increased expression of *NbHAOX8* and *NbGOX1* genes (Fig. 6B), indicating that *NbGOX4* might function through repressing *NbHAOX8* and *NbGOX1*. This result coincides with the obersavations that *NbHAOX8* and *NbGOX1* often function similarly in H_2_O_2_ accumulation and diseasae resistance in opposite to *NbGOX4*. Moreover, interactions between members of GOX family have been reported for isozymes of rice GLOs^6^.

## Conclusions

Glycolate oxidase (GOX) gene family in *Nicotiana benthamiana* was identified at genome-wide level and their function in disease resistance was analyzed. *N*. *benthamiana* genome contained 16 *GOX* genes, which was much more than Arabidopsis and rice. The alpha_hydroxyacid_oxid_FMN domain in all but two currently identified GOX family proteins contained an extra 43–52 amino acids compared with that of FMN-dependent alpha-hydroxyacid oxidizing enzymes deposited in NCBI-CDD (cd02809), and may represent a characteristic feature of plant GOX subfamily of these enzymes. Three *NbGOX* family genes *NbHAOX8*, *NbGOX1* and *NbGOX4* played distinct roles in nonhost resistance to *Xanthomonas oryzae* pv. *oryzae*, host resistance to *Sclerotinia sclerotiorum* and flg22-triggered immunity. This reveals that GOX family genes are involved in various types of disease resistance and this function of GOX family genes may differ up to plant species, gene members, types of resistance and pathogens/elicitors. These NbGOX family genes altered resistance via modulate H_2_O_2_ accumulation. Additionally, members of NbGOX family involved distinct defense pathways to affect disease resistance, and *NbGOX4* may function through repressing *NbHAOX8* and *NbGOX1*. Collectively, our results reveal that NbGOX family genes play important roles in various types of disease resistance and functions and mechanisms of family members in resistance are distinguishable.

## Electronic supplementary material

Supplementary Materials
