# Supplementary material for: Glycolate oxidase gene family in Nicotiana benthamiana: genome-wide identification and functional analyses in disease resistance
Source: Sci Rep. 2018 Jun 5;8:8615. doi: 10.1038/s41598-018-27000-4 (PMC5988680; doi:10.1038/s41598-018-27000-4)
Supplement: Supplementary file 1 — Supplementary Materials [file 41598_2018_27000_MOESM1_ESM.pdf]

Supplementary Materials

Glycolate oxidase gene family in *Nicotiana benthamiana*: genome-wide identification and functional analyses in disease resistance

You-Ping Xu[1,2], Juan Yang[1] & Xin-Zhong Cai[1,*]

[1] State key laboratory of Rice Biology, Institute of Biotechnology, College of Agriculture and Biotechnology, Zhejiang University, 866 Yu Hang Tang Road, Hangzhou 310058, China

[2] Centre of Analysis and Measurement, Zhejiang University, 866 Yu Hang Tang Road, Hangzhou 310058, China

Supplementary Table

Supplementary Table S1 Primers used in this study

Gene	Primer name	Sequence (5’→3’)	Product (bp)
Primers for qRT-PCR			
Nb18srDNA	Nb18srDNA-F	AGGATTGACAGACTGAGAGC	210
	Nb18srDNA-R	CACAGACCTGTTATTGCCTC	
TRV1 Rep	TRV1 Rep-F	ATCTCAAGTTGATTTGAGGTT	461
	TRV1 Rep-R	TGATCTCTTTGCTTACATCGT	
TRV2 2b	TRV2 2b-F	ATGCACGAATTACTTAGGAAG	303
	TRV2 2b-R	GGTAACCTTACTCACAGAAT	
NbHAOX8	NbHAOX8-F	TAGTTTCGTCGTGGAAGCGG	212
	NbHAOX8-R	TAAAGGGGAAGCAGAAATCA	
NbGOX1	NbGOX1-F	GCAGCCAGCAAGAGAAGTCA	132
	NbGOX1-R	TCAATGTAACGGCAAAAAGAG	
NbGOX4	NbGOX4-F	GTGAAGGGAAACTGTGAAAC	140
	NbGOX4-R	GGTGGGGTGTGGATGAAGA	
NbPR1	NbPR1-F	GTGCCCAAAATTCTCAACA	196
	NbPR1-R	AAATCGCCACTTCCCTCAG	
NbNPR1	NbNPR1-F	CGCCGGCGGAGATTACTTCACT	163
	NbNPR1-R	GGACTCCTCGCCGACAAAATG	
NbRbohD	NbRbohD-F	AACCGGCGCCAAGTCCAATGATG	236
	NbRbohD-R	TTAAAGAAGCCAAACGCCTGAGC	
NbRbohF	NbRbohF-F	CAGATTCTTCCGGTCAGGCTTGTA	249
	NbRbohF-R	ACCCGCTTGAGTTCTTGAGTCCAG	
NbPDF1.2	NbPDF1.2-F	ACAGAGATGGGACCAACGACA	160
	NbPDF1.2-R	AGAAACAACGGCGGCGGAAT	
Nbwrky45	Nbwrky45-F	GAGAGAATCTGCAACAGTGG	181
	Nbwrky45-R	GTATGTGGTCTGGTAAATGA	
Nbwrky62	Nbwrky62-F	GGATGATACTCCACCTACTA	195

	Nbwrky62-R	CCCTCTCCACATGCTTCTTC	
Primers for making VIGS constructs			
NbHAOX8	NbHAOX8-F	ggatccGCATAGCTCCAACTCTACAA	237
	NbHAOX8-R	gaattcATATCAGATTGCTCGTGACT	
NbGOX1	NbGOX1-F	ggatccATATCGTTGCCTCCTATGAG	243
	NbGOX1-R	gaattcAAGAACTTCACCTTACACCTG	
NbGOX4	NbGOX4-F	ggatccAACCATCCATTTGCCCTTCTA	334
	NbGOX4-R	gaattcGCAATCGCCTCATACTCCAT	

Supplementary Figures

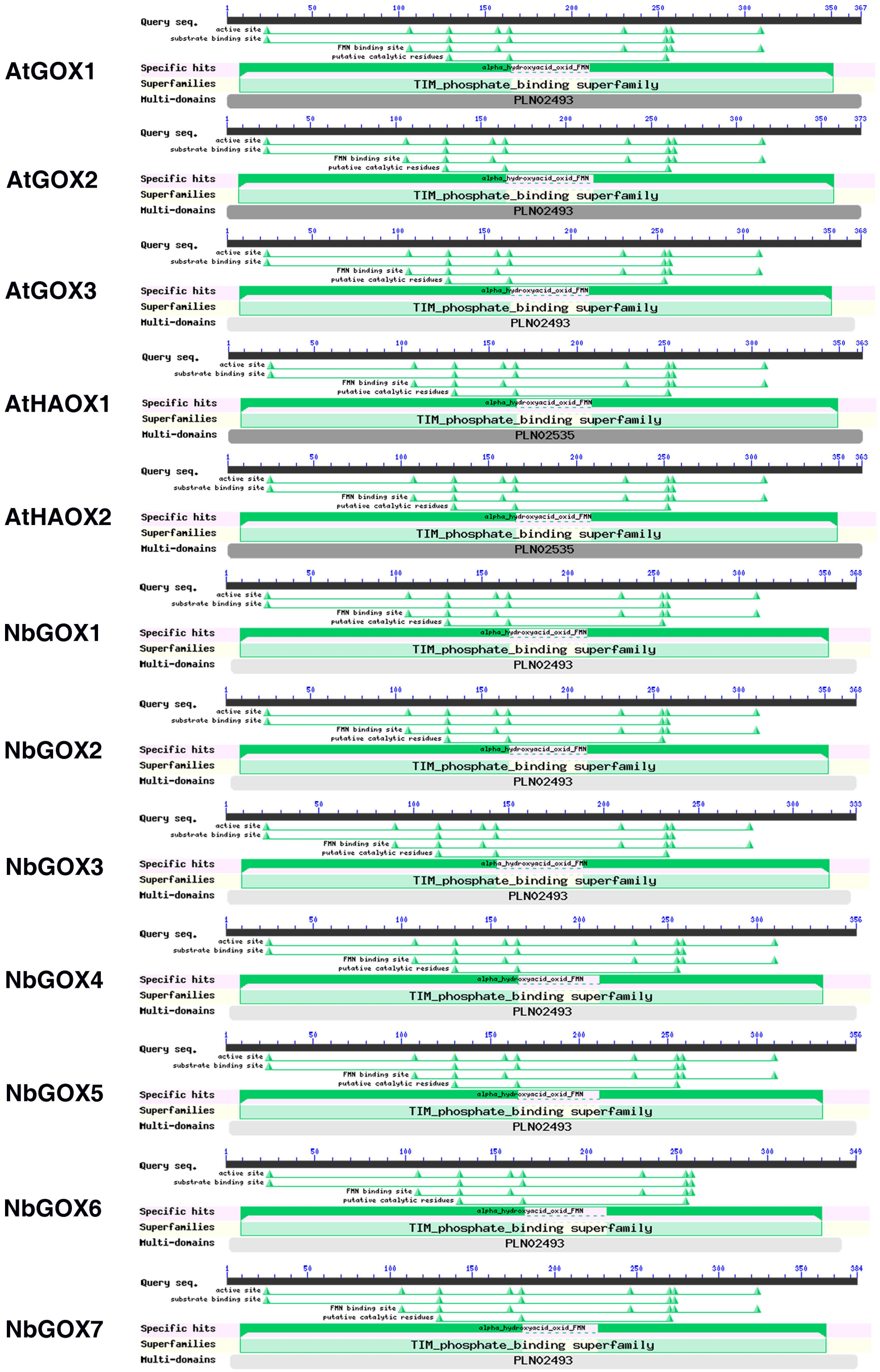

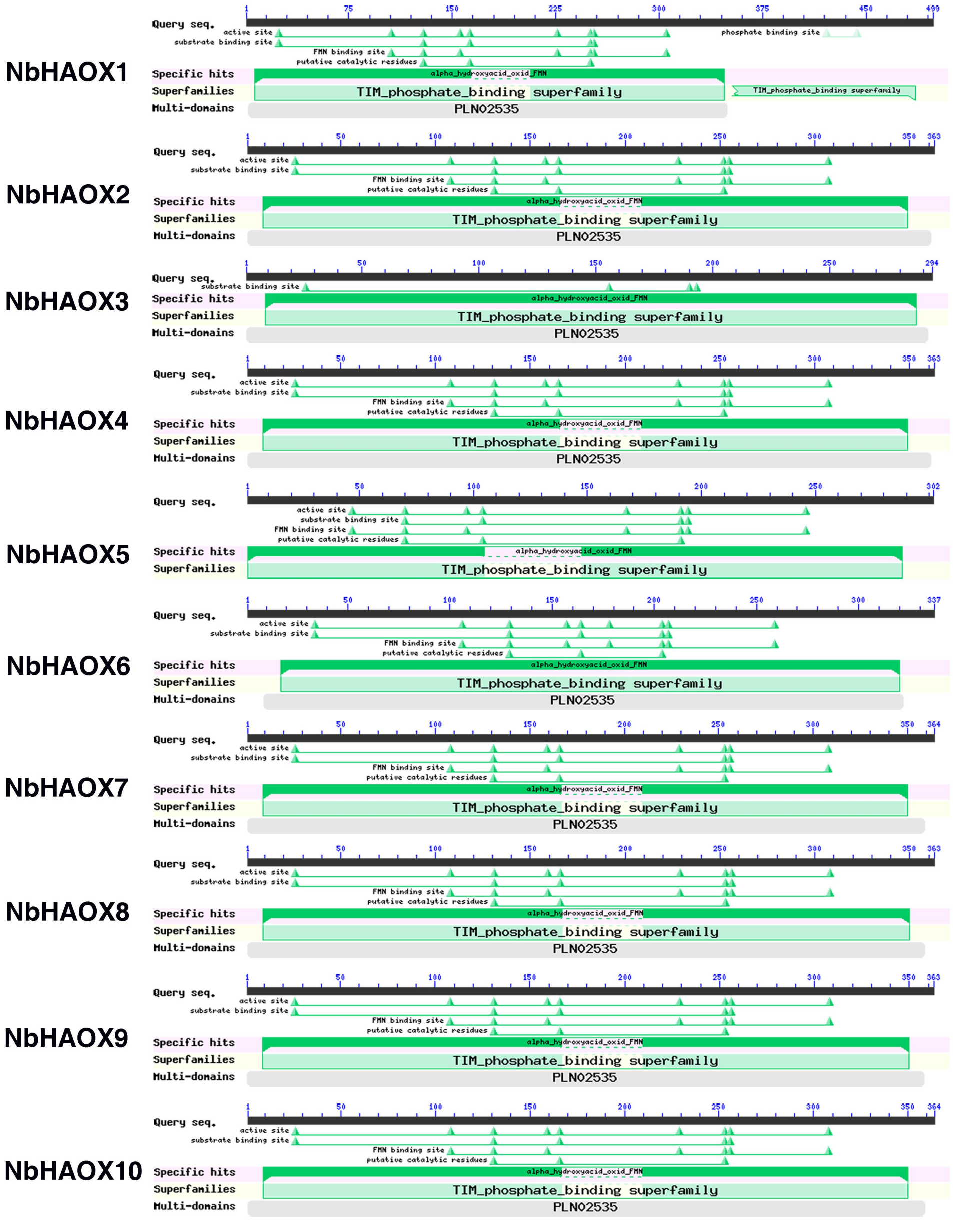

Supplementary Figure S1. Domain composition of GOX family proteins in *Nicotiana benthamiana* and *Arabidopsis thaliana*. Domain composition analysis was performed online using NCBI-CDD program.

Percent Identity

Divergence

	1	2	3	4	5	6	7	8	9	10	11	12	13	14	15	16	17	18	19	20	21	22		
**1**		95.1	86.4	61.6	59.7	85.6	85.3	75.0	87.8	87.5	90.1	88.8	54.7	54.8	51.4	55.1	56.0	59.7	59.4	60.1	59.8	59.9	**1**	AtGOX1
**2**	5.1		84.0	61.2	59.8	83.1	82.8	72.6	86.1	85.8	88.7	87.9	54.7	54.8	51.7	55.1	56.3	59.5	59.9	60.1	59.3	59.4	**2**	AtGOX2
**3**	15.1	18.1		61.7	60.6	85.0	84.7	72.6	81.9	81.6	86.1	83.9	53.1	52.6	49.7	52.9	53.3	57.5	57.7	58.7	59.0	58.6	**3**	AtGOX3
**4**	53.4	54.2	53.1		91.8	61.7	61.4	55.8	59.8	59.5	61.9	59.8	66.4	66.6	60.4	67.4	67.5	69.7	70.8	71.3	73.2	73.0	**4**	AtHAOX1
**5**	57.2	57.0	55.3	8.7		59.5	59.2	54.5	58.6	58.3	60.7	58.7	66.9	67.1	61.1	67.7	66.6	69.4	70.5	69.6	71.8	71.6	**5**	AtHAOX2
**6**	16.1	19.2	16.8	53.1	57.6		99.5	78.7	84.1	83.9	85.8	83.6	53.9	53.6	51.2	54.1	55.0	59.2	58.4	59.9	59.7	59.5	**6**	NbGOX1
**7**	16.4	19.5	17.1	53.7	58.2	0.5		78.4	83.9	83.6	85.5	83.3	53.6	53.3	50.9	53.9	54.6	58.8	58.1	59.7	59.4	59.2	**7**	NbGOX2
**8**	30.4	34.1	34.1	65.7	68.5	25.1	25.6		73.3	73.0	75.2	73.1	49.8	49.5	45.4	50.2	50.7	54.6	54.8	55.0	54.4	55.2	**8**	NbGOX3
**9**	13.4	15.4	20.8	57.0	59.4	17.9	18.2	33.1		99.7	92.0	89.0	52.8	52.7	49.3	52.7	53.7	56.0	56.3	57.1	58.2	58.6	**9**	NbGOX4
**10**	13.7	15.8	21.2	57.6	60.0	18.2	18.6	33.6	0.3		91.7	88.8	52.8	52.7	49.3	52.7	53.7	56.0	56.3	57.1	58.2	58.6	**10**	NbGOX5
**11**	10.6	12.3	15.4	52.8	55.1	15.7	16.1	30.1	8.5	8.8		96.6	55.0	54.4	51.3	54.7	54.6	59.9	58.4	60.3	60.9	60.4	**11**	NbGOX6
**12**	12.2	13.2	18.2	56.9	59.2	18.5	18.9	33.3	11.9	12.2	3.5		53.4	52.8	48.8	53.1	53.0	58.2	57.3	58.9	59.2	58.4	**12**	NbGOX7
**13**	68.1	68.1	72.1	44.4	43.4	70.0	70.7	80.3	72.8	72.8	67.4	71.3		98.9	84.2	95.0	91.7	79.5	85.0	84.2	78.8	79.4	**13**	NbHAOX1
**14**	67.8	67.8	73.1	44.1	43.1	70.8	71.4	81.1	72.8	72.8	68.8	72.7	1.1		84.4	96.1	92.1	80.1	85.4	83.1	77.9	79.9	**14**	NbHAOX2
**15**	76.3	75.4	80.8	55.7	54.4	76.7	77.6	93.1	81.8	81.8	76.5	83.1	17.7	17.6		81.6	80.3	75.4	77.2	76.9	74.1	76.2	**15**	NbHAOX3
**16**	67.1	67.1	72.4	42.7	42.2	69.4	70.1	79.4	72.8	72.8	68.1	72.1	5.2	4.0	21.1		91.4	84.6	87.3	85.9	77.9	82.4	**16**	NbHAOX4
**17**	65.2	64.5	71.4	42.4	44.1	67.5	68.3	78.0	70.6	70.6	68.3	72.2	8.8	8.4	23.0	9.2		80.0	88.1	85.0	79.1	82.1	**17**	NbHAOX5
**18**	57.2	57.6	61.8	38.8	39.3	58.3	59.0	68.4	65.1	65.1	56.9	60.3	24.0	23.2	29.9	17.2	23.3		90.6	87.6	78.1	82.7	**18**	NbHAOX6
**19**	57.8	56.7	61.3	37.0	37.4	59.9	60.5	67.8	64.4	64.4	60.0	62.2	16.8	16.3	27.2	13.9	13.0	10.1		91.5	82.4	87.4	**19**	NbHAOX7
**20**	56.3	56.3	59.2	36.2	38.9	56.7	57.2	67.4	62.8	62.8	56.0	58.9	17.8	19.1	27.7	15.6	16.7	13.6	9.1		86.2	85.4	**20**	NbHAOX8
**21**	56.9	58.0	58.6	33.2	35.3	57.2	57.8	68.8	60.3	60.3	54.8	58.3	24.9	26.2	31.7	26.2	24.6	25.9	20.2	15.3		94.5	**21**	NbHAOX9
**22**	56.7	57.8	59.5	33.5	35.6	57.6	58.2	67.1	59.4	59.4	55.7	59.8	24.1	23.5	28.7	20.2	20.5	19.7	13.9	16.3	5.7		**22**	NbHAOX10
	**1**	**2**	**3**	**4**	**5**	**6**	**7**	**8**	**9**	**10**	**11**	**12**	**13**	**14**	**15**	**16**	**17**	**18**	**19**	**20**	**21**	**22**		

Supplementary Figure S2. Sequence similarity of GOX family proteins in *Nicotiana benthamiana* and *Arabidopsis thaliana.* Sequence comparison was performed using MegAlign program of Lasergene software package.
